# Supplementary material for: Hydrogenation of benzoic acid derivatives over Pt/TiO2 under mild conditions
Source: Commun Chem. 2021 Apr 16;4:54. doi: 10.1038/s42004-021-00489-z (PMC9814562; doi:10.1038/s42004-021-00489-z)
Supplement: Supplementary file 1 — Supplementary Infromation [file 42004_2021_489_MOESM1_ESM.pdf]

Supporting Information

Hydrogenation of benzoic acid derivatives over Pt/TiO_2 under Mild Conditions

Miao Guo[1], Xiangtao Kong[2], Chunzhi Li[1,3], Qihua Yang[1*]

[1] State Key Laboratory of Catalysis, Dalian Institute of Chemical Physics, Chinese Academy of Sciences, Dalian 116023, China.

[2] College of Chemistry and Chemical Engineering, Anyang Normal University, Anyang 455000, China.

[3] University of Chinese Academy of Sciences, Beijing 100039, China.

Email: yangqh@dicp.ac.cn;

Table of Contents

1. Mass transfer calculations 3
2. Additional catalytic results and characterization information 5
Supplementary Figure 1 5
Supplementary Figure 2 5
Supplementary Figure 3 6
Supplementary Figure 4 6
Supplementary Figure 5 7
Supplementary Figure 6 7
Supplementary Figure 7 8
Supplementary Figure 8 8
Supplementary Figure 9 9
Supplementary Figure 10 9
Supplementary Figure 11 10
Supplementary Figure 12 10
Supplementary Figure 13 11
Supplementary Figure 14 11
Supplementary Figure 15 12
Supplementary Figure 16 12
Supplementary Figure 17 13

Supplementary Figure 18 13
Supplementary Figure 19 14
Supplementary Figure 20 14
Supplementary Figure 21 15
Supplementary Figure 22 15
Supplementary Figure 23 16
Supplementary Figure 24 16
Supplementary Figure 25 17
Supplementary Figure 26 17
Supplementary Figure 27 18
Supplementary Table 1 19
Supplementary Table 2 19
Supplementary Table 3 20
Supplementary Table 4 20
Supplementary Table 5 20
Supplementary References 21

Supplementary Methods

1. Mass transfer calculations

To ensure the collection of reliable kinetic data, mass transfer tests were conducted. A control experiment was first carried out at different stirring rates and the results showed that the reaction rate remained constant in the range of 750–1200 rpm. The reactions in the study were conducted under 1000 rpm in order to avoid the catalyst splashing.

Furthermore, the widely used criteria of mass transfer tests in three phases stirring reactors were used[1]. The criteria, which define the ratio of observed rate to the maximum rate, are shown as followed:

$$\alpha_1 = \frac{r_A}{k_L a_B C_{A*}} < 0.1 \quad \text{Supplementary Equation 1}$$

$$\alpha_2 = \frac{r_A}{k_s a_p C_{A*}} < 0.1 \quad \text{Supplementary Equation 2}$$

where α_1 describes the gas-liquid mass transfer ratio, and α_2 describes the liquid-solid mass transfer ratio, r_A is the initial reaction rate of BA hydrogenation (0.213 mol L^{-1} h^{-1}), $k_L a_B$ is the gas-liquid mass transfer coefficient (Eq. 3), C_A* is the saturation solubility in hexane that can be obtained from ref. 2 (0.075 kmol m^{-3}, 298.15 K, 1.0 MPa H_2), k_s is the effective diffusivity (Eq. 4), and a_p is the catalyst external surface area (29 m^2 g^{-1}). If each requirement is met ($\alpha_1 < 0.1$, $\alpha_2 < 0.1$,), it can be said that the system is free of gas-liquid and liquid-solid mass transfer.

The $k_L a_B$ in Eq. 1 can be calculated as follows[3]:

$$k_L a_B = 1.48 \times 10^{-3} (\text{N})^{2.18} (\frac{V_g}{V_L})^{1.88} (\frac{d_I}{d_T})^{2.16} (\frac{h_1}{h_2})^{1.16} \quad \text{Supplementary Equation 3}$$

The values used here are: N, the stirring speed, is 16.7 Hz; V_g, the volume of H_2, is 3 × 10^{-4} m^3; V_L, the volume of liquid, is 3 × 10^{-4} m^3; d_I, the diameter of the impeller, is 0.01 m; d_T, the diameter of the reactor tank, 0.015 m; h_1, the height of the impeller from the bottom of the tank, is 0.003 m; h_2, he height of the liquid in the tank, is 0.02 m. Thus, the $k_L a_B$ value is 0.03 s^{-1}. Applying r_A, $k_L a_B$ and C_A* in Eq. 1 gives α_1 of 0.02, which is well below 0.1, indicating the system is not limited by the rate of gas to liquid mass transfer.

The liquid-solid mass transfer coefficient (ks) in Eq. 2 can be calculated from the Sherwood number (Sh) in Eq. 4 (refs. 4,5):

$$\text{Sh} = [2 + 0.4(\frac{\varepsilon d_p{}^4}{v^3})^{\frac{1}{4}} \text{Sc}^{\frac{1}{3}}] \Phi_c = \frac{k_s k_p}{D_A} \quad \text{Supplementary Equation 4}$$

The right side of Eq.4: d_p, the specific surface diameter, can be obtained from the density (4.26 × 10^6 g m^{-3}) and the BET surface area of rutile (29 m^2 g^{-1}) [4,5]. The calculated value of d_p is 4.86 × 10^{-8} m. D_A is the diffusivity of hydrogen in hexane. The experimental value of D_A is 62.38 × 10^{-9} m^2 s^{-1} (298K)[6].

The left side of Eq.4: ν is the kinematic viscosity of hexane. The experimental value of ν is 4.247 × 10^{-7} m^2 s^{-1} (313K). The Schmidt number (Sc) is can be calculated from the ratio of ν/D_A = 6.8 (ref. 7). Φ_c is the Carman's surface factor, which can be

calculated from the d_p/d_p'. The approximate value of d_p' (the screen diameter) is $2d_p$ (refs. 4,5). Thus, $\Phi_c \approx 0.5$. ε is the rate of flow energy supply per unit mass of liquid, which can be calculated from Eq. 5.

$$\varepsilon = \frac{N_p l^5 n^3}{V_L} \quad \text{Supplementary Equation 5}$$

where Np is the impeller power number. $N_p = 3$ for the reaction system[8]. l is the diameter of impeller (0.03 m), n is the rps of impeller (105 rad s^{-1}), and V_L is the volume of liquid (3×10^{-4} m^3). Eq. 5 gives $\varepsilon = 281$ m^2 s^{-3}. Combining the above data and Eq. 4, k_s with the value of 2.58 m s^{-1} can be calculated. From Eq. 2, a_p is the external surface area of the catalyst per unit volume of reactor and is calculated to be 1.03×10^6 m^2 m^{-3}. Using these values obtained and plugging into Eq. 2 yields an $\alpha_2 = 2.9\times10^{-4}$, which is orders of magnitude below 0.1 indicating the system is not mass transfer limited in the liquid-solid regime.

The above results show the kinetic data were in the absence of external mass transfer limitation. Finally, the low surface area and the extremely low pore volume of the TiO_2 diminish the presence of intra-particle mass transfer limitation.

2. Additional catalytic results and characterization information

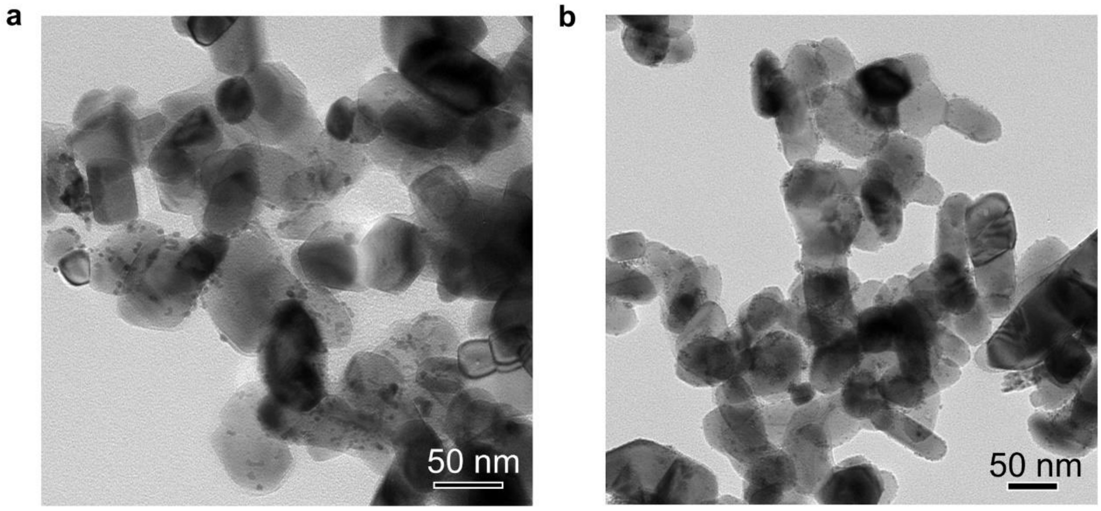

Supplementary Figure 1. TEM images of **a** Pd/TiO_2 and **b** Ru/TiO_2.

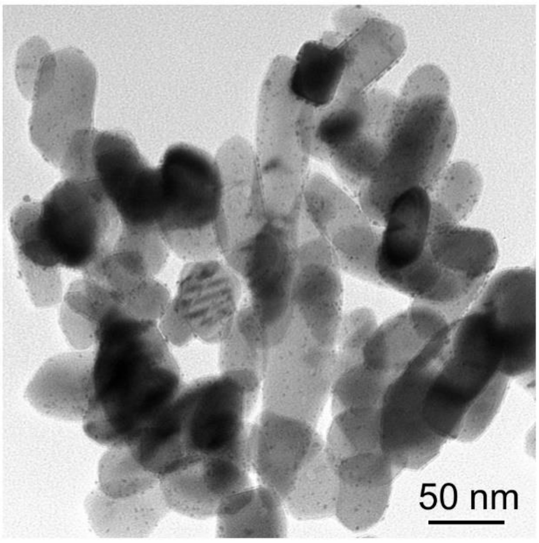

Supplementary Figure 2. TEM image of Pt/TiO_2.

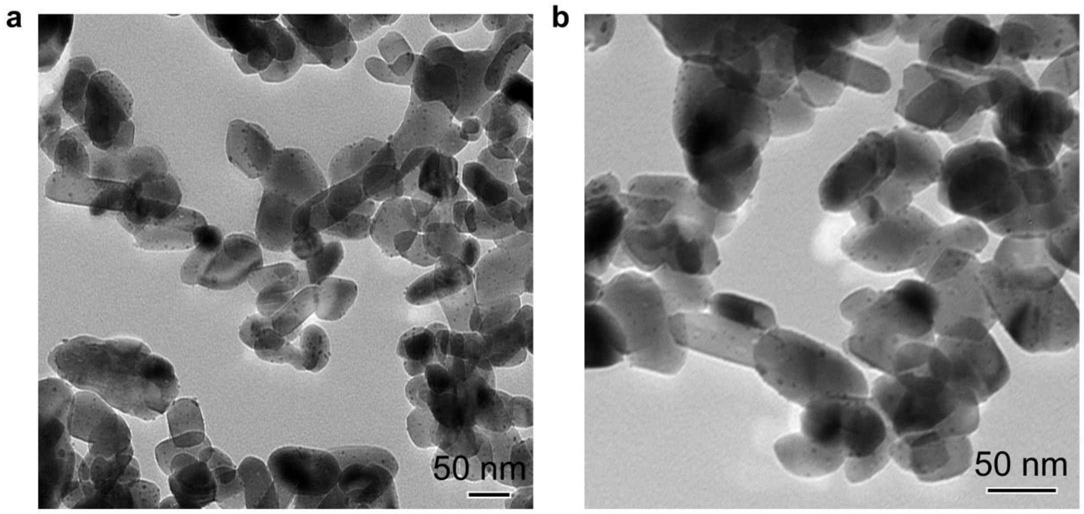

Supplementary Figure 3. TEM images of **a** Pt/TiO_2-200 and **b** Pt/TiO_2-450.

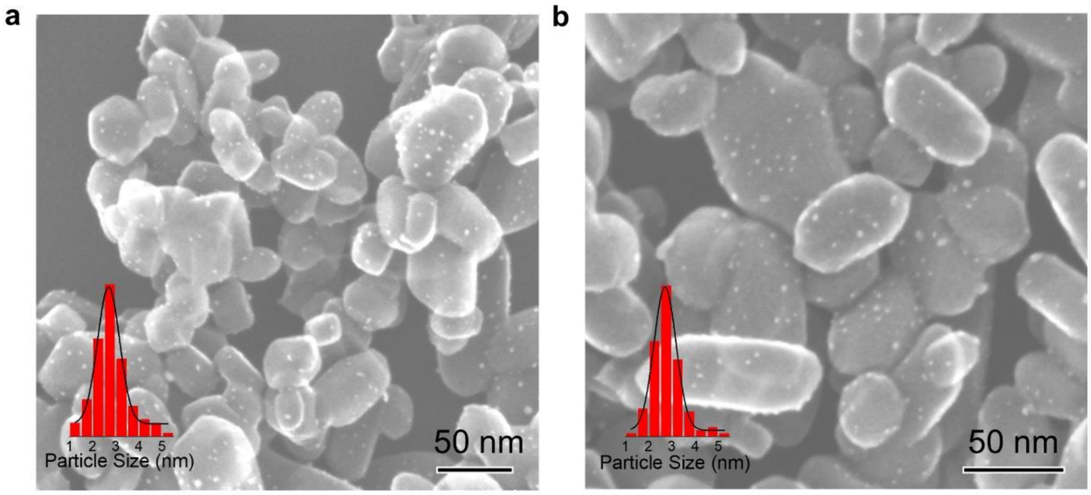

Supplementary Figure 4. HRSEM images of **a** Pt/TiO_2-200 and **b** Pt/TiO_2-450.

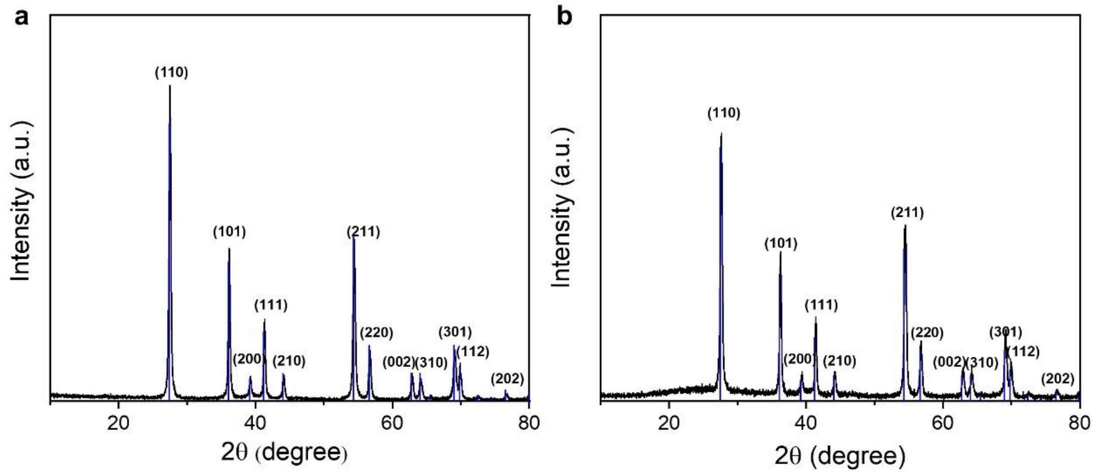

Supplementary Figure 5. XRD patterns of **a** Pt/TiO_2 and **b** Pt/TiO_2-450.

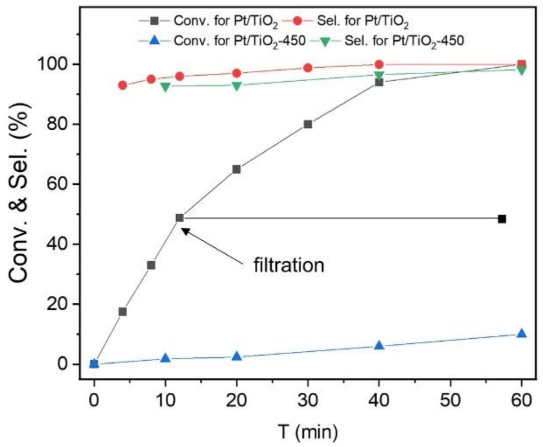

Supplementary Figure 6. Reaction profiles of Pt/TiO_2 and Pt/TiO_2-450. Reaction conditions: 40 °C, 10 bar H_2, 0.12 mmol BA, S/C=250, 3 mL hexane.

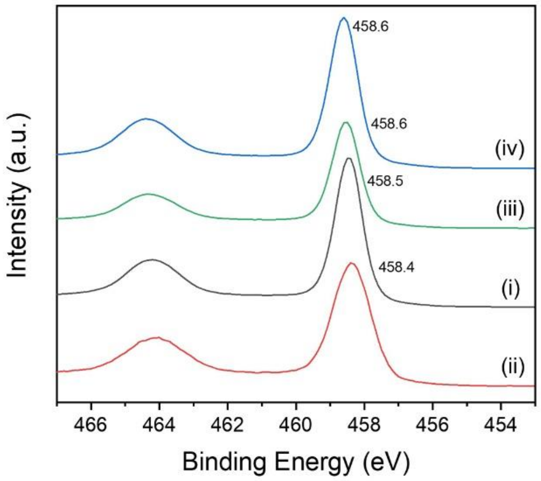

Supplementary Figure 7. XPS spectra of Ti 2p of **i** TiO_2, **ii** Pt/TiO_2, **iii** Pt/TiO_2-200 and **iv** Pt/TiO_2-450.

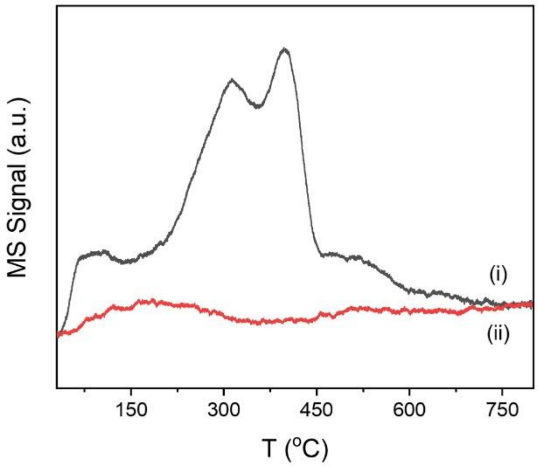

Supplementary Figure 8. H_2-TPD profiles of **i** Pt/TiO_2 and **ii** Pt/TiO_2-450.

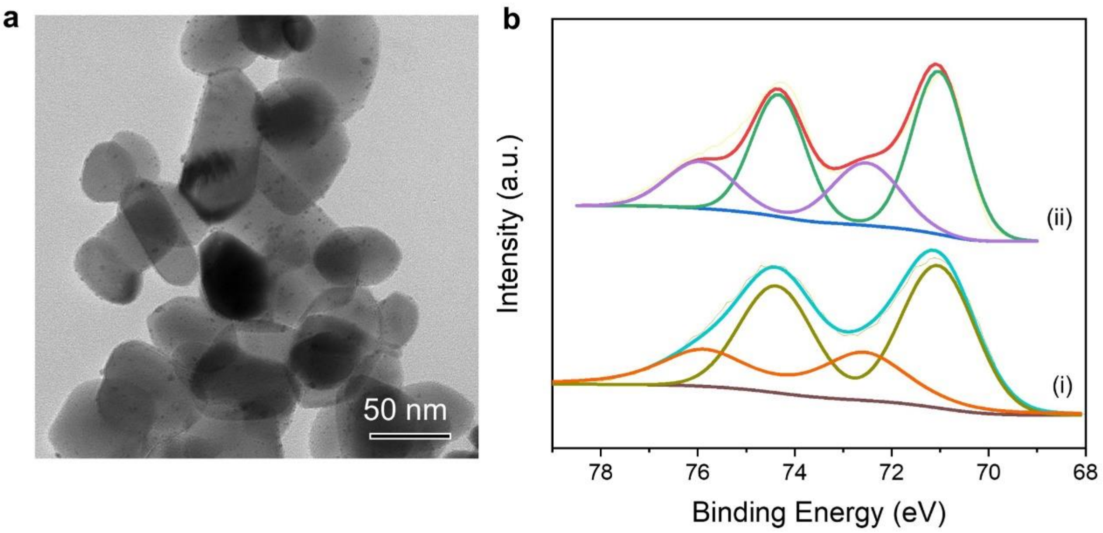

Supplementary Figure 9. **a** TEM image of Pt/TiO_2 after 5th recycle. **b** XPS spectra of Pt 4f of **i** Pt/TiO_2 and **ii** Pt/TiO_2 after 5th recycle.

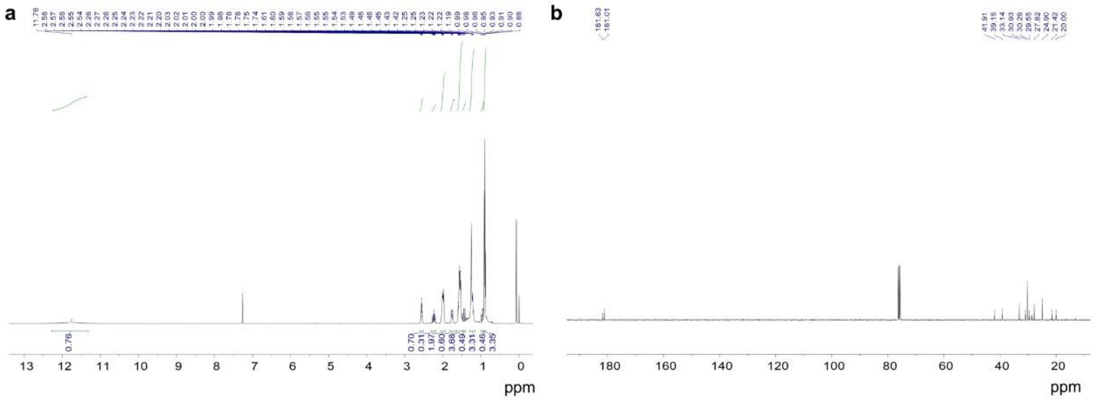

Supplementary Figure 10. **a** ^{1}H NMR and **b** ^{13}C NMR spectra of 4-Methylcyclohexanecarboxylic acid.

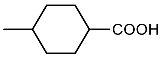

^{1}H NMR (400 MHz, $CDCl_3$): δ 11.76 (s, 1H), 2.56 (m, J = 4.8 Hz, 0.7H), 2.24 (tt, J = 12.4, 3.6 Hz, 0.3H), 2.03-1.98 (m, 2H), 1.78-1.19 (m, 7H), 0.9 (t, 6 Hz, 3H) ppm, (cis:trans = 70:30). ^{13}C NMR (100 MHz, $CDCl_3$): δ 181.63, 181.01, 41.91, 39.15, 33.14, 30.93, 30.29, 29.55, 27.82, 24.90, 21.42, 20.00 ppm.

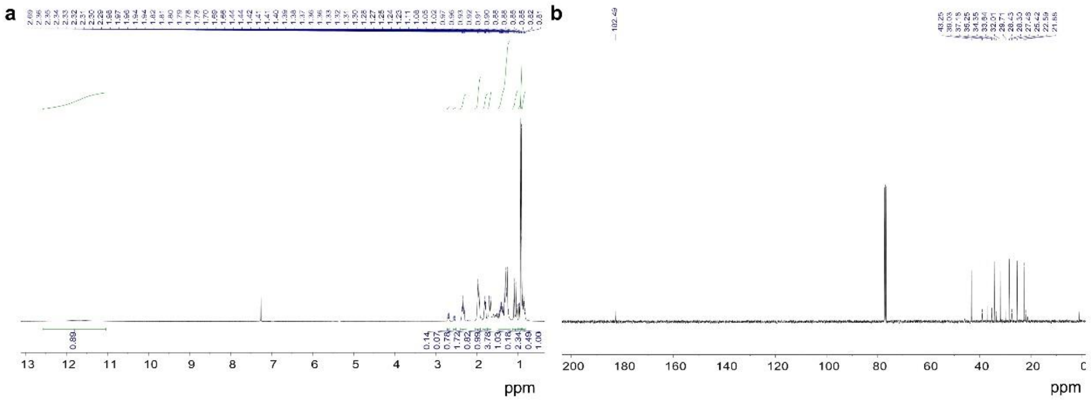

Supplementary Figure 11. **a** ^{1}H NMR and **b** ^{13}C NMR spectra of 3-Methylcyclohexanecarboxylic acid.

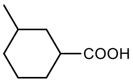

^{1}H NMR (400 MHz, $CDCl_3$): δ 11.71 (s, 1H), 2.712-2.520 (m, J =4.8 Hz, 0.21H), 2.333 (tt, J = 12, 3.6 Hz, 0.79H), 1.98-1.94 (2H, m), 1.82-1.65 (m, 2H), 1.45-0.81 (9H, m) ppm, (cis:trans=79:21). ^{13}C NMR (100 MHz, $CDCl_3$): δ 182.49, 43.25, 39.03, 37.18, 35.25, 34.35, 33.64, 32.01, 29.71, 28.43, 28.30, 27.46, 25.42, 22.59, 21.88 ppm.

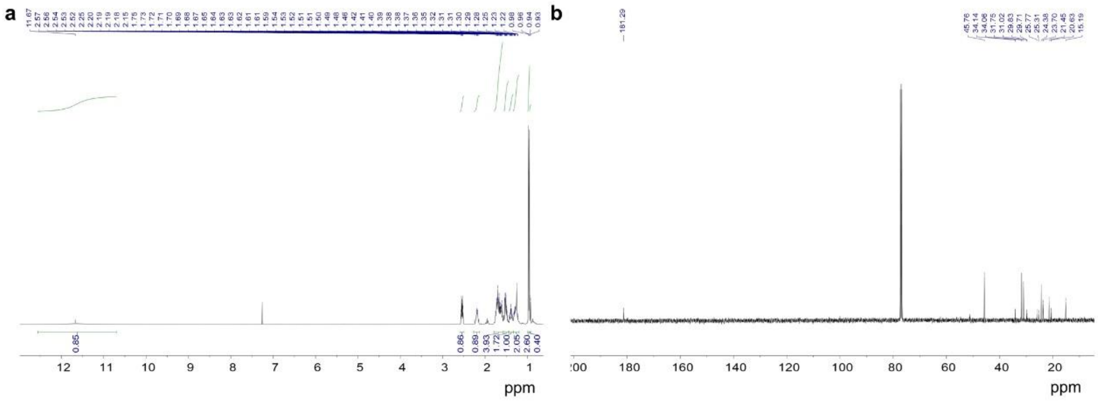

Supplementary Figure 12. **a** ^{1}H NMR and **b** ^{13}C NMR spectra of 2-Methylcyclohexanecarboxylic acid.

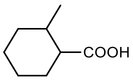

^{1}H NMR (400 MHz, $CDCl_3$): δ 11.67 (s, 1H), 2.57-2.15 (2 m, H), 1.75-1.22 (m, 9H), 0.98-0.96 (d, 7.2 Hz, 2.6H), 0.94-0.93 (d, 6.8 Hz, 0.4 H) ppm, (cis:trans=86:14). ^{13}C NMR (100 MHz, $CDCl_3$): δ 181.29, 45.76, 34.14, 34.06, 31.75, 31.02, 29.83, 29.71, 25.77, 25.31, 24.38, 23.70, 21.45, 20.63 ppm.

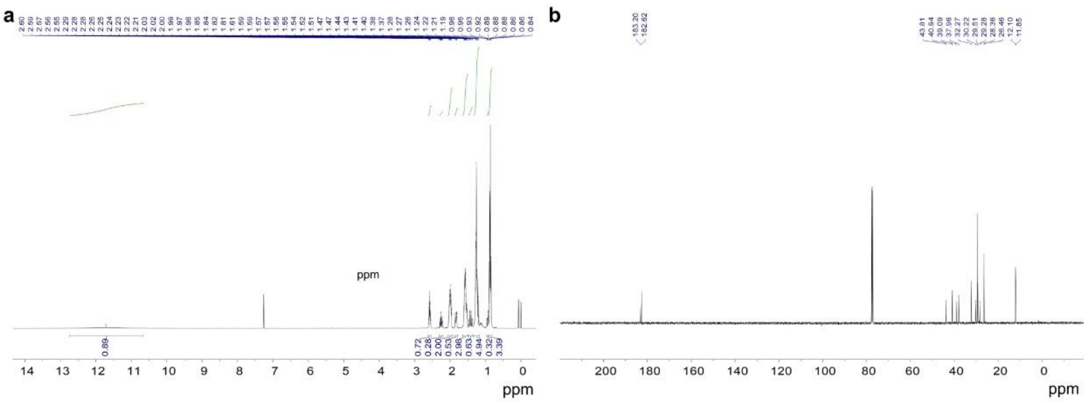

Supplementary Figure 13. **a** ^{1}H NMR and **b** ^{13}C NMR spectra of 4-Ethylcyclohexanecarboxylic acid.

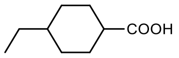

^{1}H NMR (400 MHz, $CDCl_3$): δ 11.71 (s, 1H,), 2.60-2.55 (m, J = 4.8 Hz, 0.72H), 2.25 (tt, J = 12.4, 3.6 Hz, 0.28H), 2.03-1.96 (m, 2H), 1.85-1.19 (m, 9H), 0.96-0.84 (m, 3H) ppm, (cia:trans=72:28). ^{13}C NMR (100 MHz, $CDCl_3$): δ 183.20, 182.62, 43.81, 40.94, 39.09, 37.96, 32.27, 30.22, 29.51, 29.28, 28.36, 26.46, 12.10, 11.85 ppm.

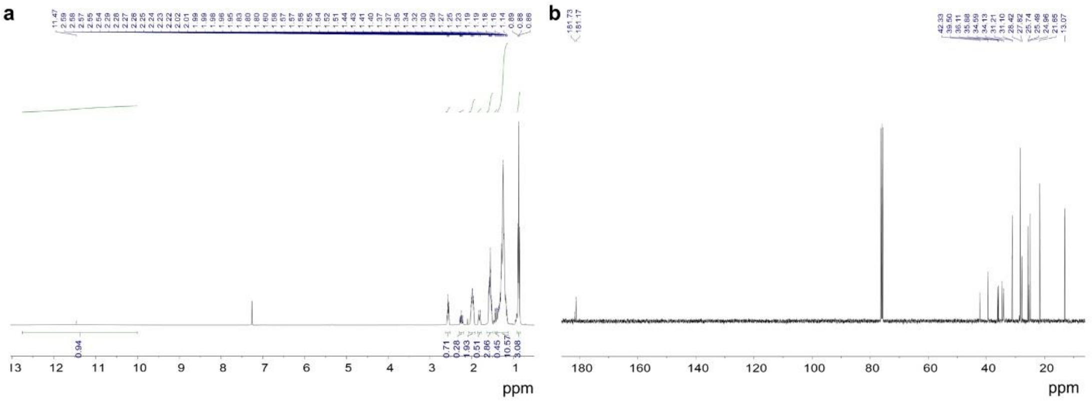

Supplementary Figure 14. **a** ^{1}H NMR and **b** ^{13}C NMR spectra of 4-Pentylcyclohexanecarboxylic acid.

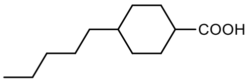

^{1}H NMR (400 MHz, $CDCl_3$): δ 11.47 (s, 1H), 2.59-2.54 (m, J = 4.8 Hz, 0.72H), 2.25 (tt, J = 12.4, 3.6 Hz, 0.28H), 2.02-1.14 (m, 17H), δ 0.88 (t, J = 6.8 Hz, 3H) ppm, (cis:trans=72:28). ^{13}C NMR (100 MHz, $CDCl_3$): δ 181.73, 181.17, 42.33, 39.50, 36.11, 35.88, 34.59, 34.13, 31.21, 31.10, 28.42, 27.82, 25.74, 25.49, 24.96, 21.65 ppm.

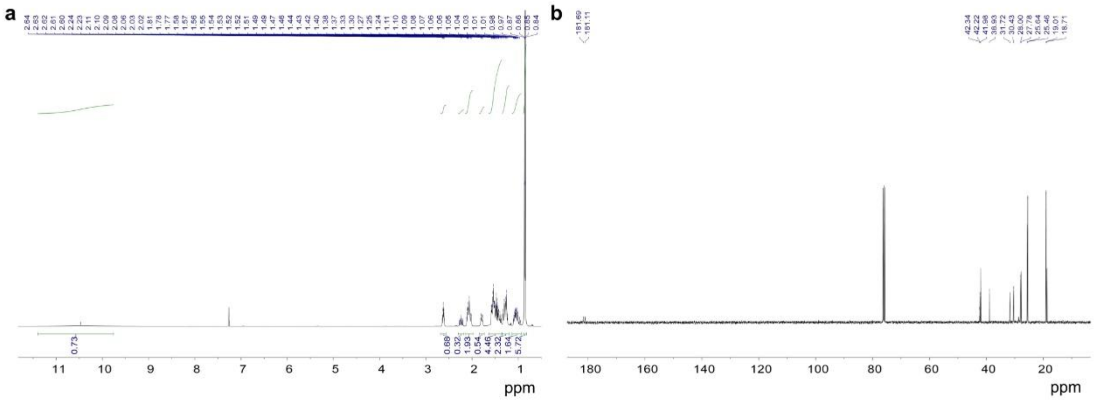

Supplementary Figure 15. **a** ^{1}H NMR and **b** ^{13}C NMR spectra of 4-Isopropylcyclohexane-1-carboxylic acid.

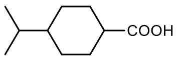

^{1}H NMR (400 MHz, $CDCl_3$): δ 10.47 (s, 1H), 2.64-2.60 (m, J = 4.4 Hz, 0.68H), 2.24 (tt, J = 12.4, 3.6 Hz, 0.32H), 2.11-2.02 (m, 2H), 1.58-0.95 (m, 9H), 0.87-0.84 (m, 6H) ppm, (cis:trans=68:32). ^{13}C NMR (100 MHz, $CDCl_3$): δ 181.69, 181.11, 42.34, 42.22, 41.98, 38.93, 31.72, 30.43, 28.00, 27.78, 25.64, 25.46, 19.01, 18.71 ppm.

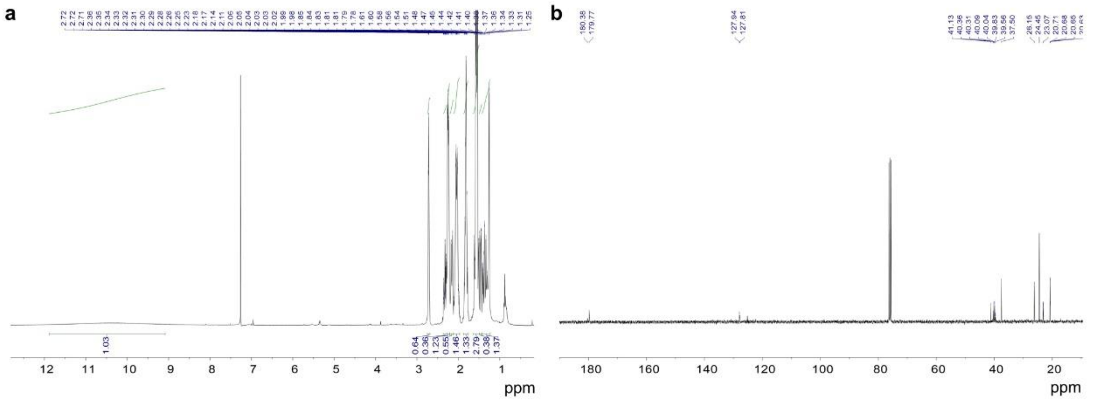

Supplementary Figure 16. **a** ^{1}H NMR and **b** ^{13}C NMR spectra of 4-Trifluoromethylcyclohexanecarboxylic acid.

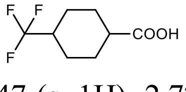

^{1}H NMR (400 MHz, $CDCl_3$): δ 10.47 (s, 1H), 2.72-2.71 (m, J = 3.2 Hz, 0.64H), 2.32 (tt, J = 12.4, 3.6 Hz, 0.36H), 2.26-1.98 (m, 3H), 1.85-1.25 (m, 6H) ppm, (cis:trans=64:36). ^{13}C NMR (100 MHz, $CDCl_3$): δ 180.38, 179.77, 127.94, 127.81, 41.13, 40.36, 40.31, 40.09, 40.04, 39.83, 39.56, 37.50, 26.15, 24.45, 23.09, 23.07, 20.71, 20.68, 20.65, 20.63 ppm.

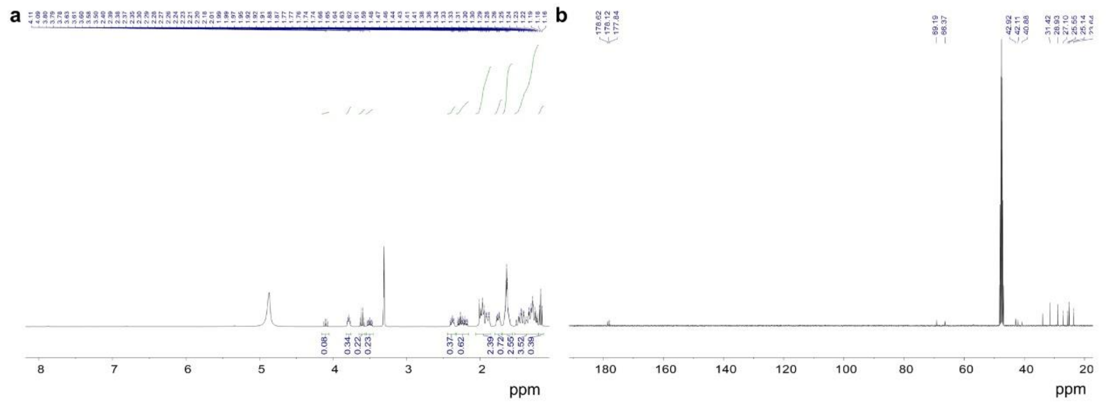

Supplementary Figure 17. **a** ^{1}H NMR and **b** ^{13}C NMR spectra of 4-Hydroxycyclohexanecarboxylic acid.

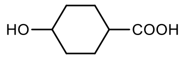

^{1}H NMR (400 MHz, $CDCl_3$): δ 8.55 (s, 1H), 4.13-4.07 (q, J = 7.2 Hz, 0.1H), 3.81-3.76 (m, J= 4.8 Hz, 0.34H), 3.63-3.58 (q, J = 7.2 Hz , 0.22H), 3.5 (tt, J = 10.8, 4 Hz, 0.23H), 2.41-2.17 (m, 1H), δ 2.01-1.16 (9H, m) ppm, (cis:trans = 60:40). ^{13}C NMR (100 MHz, $CDCl_3$): δ 178.62, 178.12, 177.84, 69.19, 66.37, 42.92, 42.11, 40.88, 33.95, 31.42, 28.93, 27.10, 25.55, 25.14, 23.64 ppm.

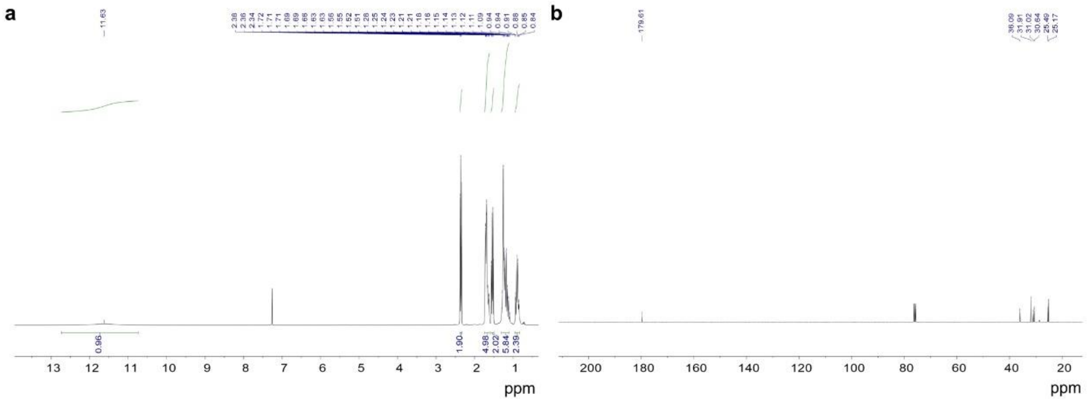

Supplementary Figure 18. **a** ^{1}H NMR and **b** ^{13}C NMR spectra of 3-Cyclohexylpropionic acid.

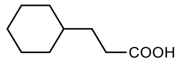

^{1}H NMR (400 MHz, $CDCl_3$): δ 11.63 (s, 1H), 2.36 (t, J = 8 Hz, 2H), 1.72-1.63 (m, 5H), 1.56-1.51 (q, J = 7.2 Hz,2H), 1.28-1.09 (m, 6H), 0.94-0.84 (m, 2H) ppm. ^{13}C NMR (100 MHz, $CDCl_3$): δ 179.6, 36.1, 31.9, 31.0, 30.6, 25.5, 25.2 ppm.

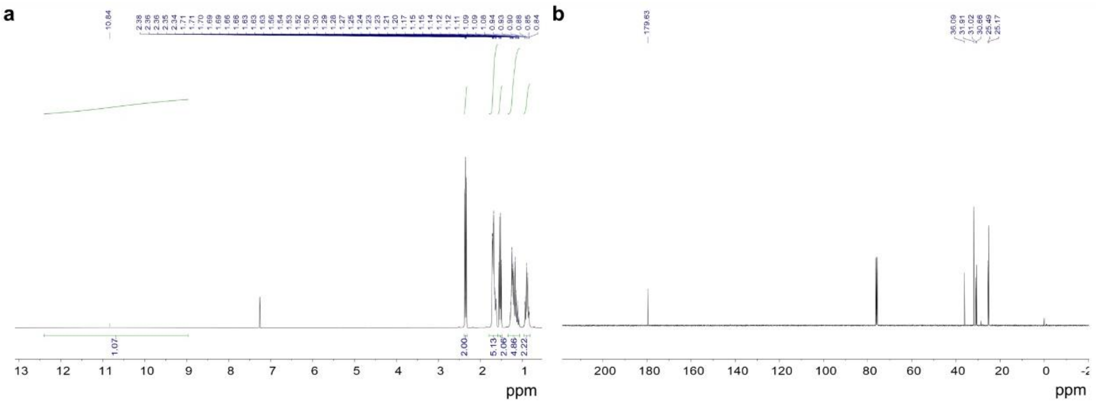

Supplementary Figure 19. **a** ^{1}H NMR and **b** ^{13}C NMR spectra of 5-cyclohexylvaleric acid.

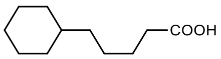

^{1}H NMR (400 MHz, $CDCl_3$): δ 10.84 (s, 1H), 2.38-2.34 (t, J = 7.2 Hz, 2H), 1.71-1.63 (m, 5H), 1.53 (q, J = 7.2 Hz, 2H), 1.30-1.08 (m, 5H), 0.94-0.84 (m, 2H) ppm. ^{13}C NMR (100 MHz, $CDCl_3$): δ 179.63, 36.09, 31.91, 31.02, 30.66, 25.49, 25.17 ppm.

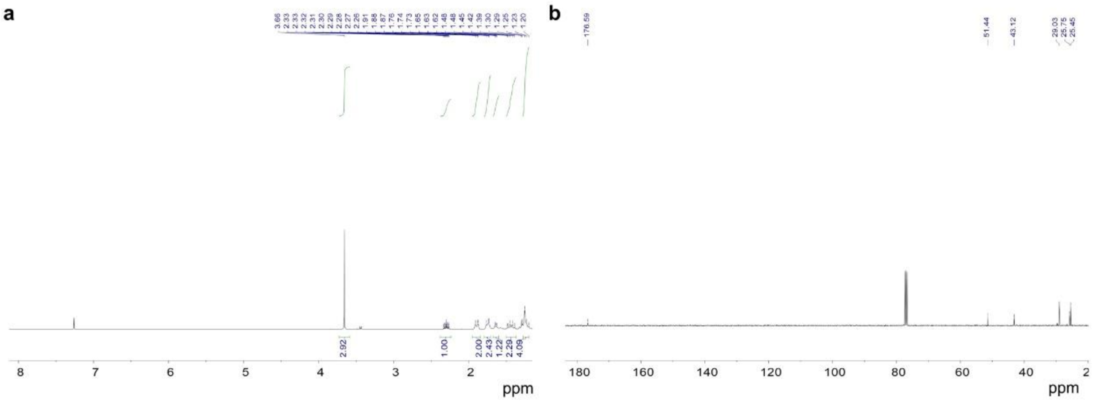

Supplementary Figure 20. **a** ^{1}H NMR and **b** ^{13}C NMR spectra of Cyclohexanecarboxylic acid methyl ester.

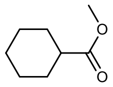

^{1}H NMR (400 MHz, $CDCl_3$): δ 3.66 (s, 3H), 2.30 (tt, J= 11.2, 3.6 Hz, 1H), 1.91-1.25 (m, 10H) ppm. ^{13}C NMR (100 MHz, $CDCl_3$): δ 176.59, 51.44, 43.12, 29.03, 25.75, 25.45 ppm.

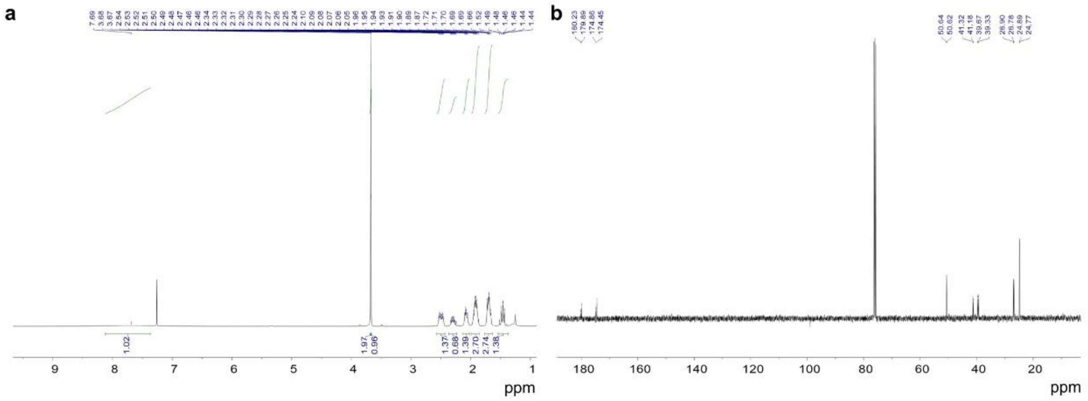

Supplementary Figure 21. **a** ^{1}H NMR and **b** ^{13}C NMR spectra of Mono-methyl terephthalate.

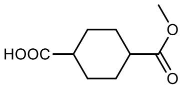

^{1}H NMR (400 MHz, $CDCl_3$): δ 7.69 (s, 1H), 3.68 (s, 2 H), 3.67 (s, 1H), 3.68-3.67 (m, 3H), 2.54-2.24 (m, 2H), 2.10-1.87 (m, 4H, m), 1.72-1.66 (m, 3H), 1.52-1.44 (m, 1H) ppm, (cis:trans = 66:34). ^{13}C NMR (100 MHz, $CDCl_3$): δ 180.23, 179.89, 174.86, 174.45, 50.64, 50.62, 41.32, 41.18, 39.67, 39.33, 26.90, 26.78, 24.89, 24.77 ppm.

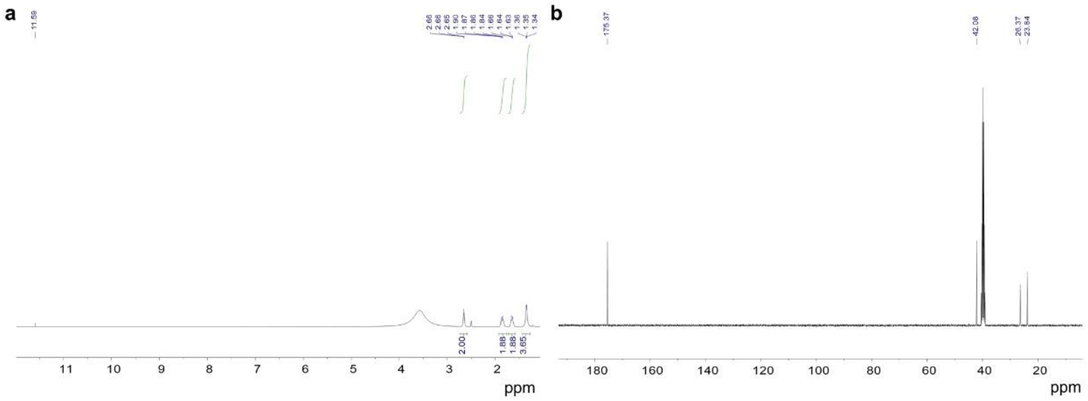

Supplementary Figure 22. **a** ^{1}H NMR and **b** ^{13}C NMR spectra of Trans-cyclohexane-1,2-dicarboxylic acid.

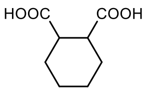

^{1}H NMR (400 MHz, DMSO-d^{6}): δ 11.59 (s, 1H), 2.66-2.65 (m, 2H), δ 1.90-1.84 (m, 2H), 1.66-1.63 (m, 2H), 1.36-1.34 (m, 4H) ppm, (cis:trans = 0:~100). ^{13}C NMR (100 MHz, DMSO-d^{6}): δ 175.4, 42.1, 26.4, 23.8 ppm.

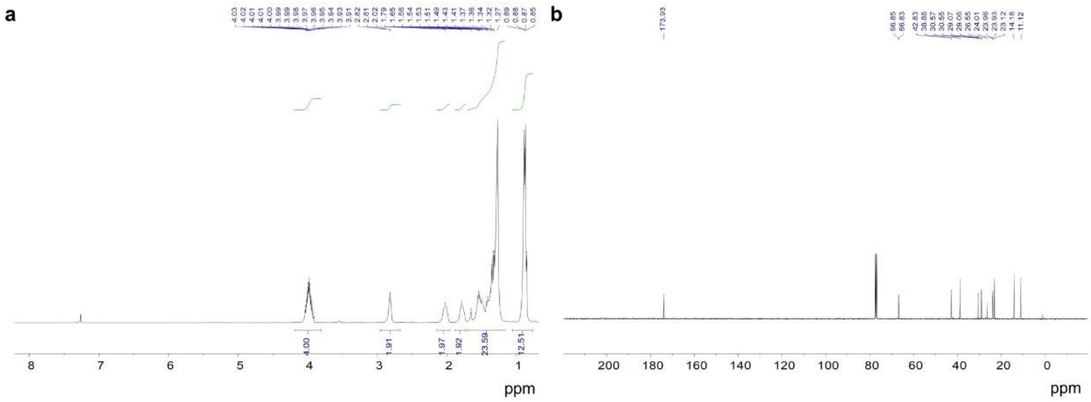

Supplementary Figure 23. **a** ^{1}H NMR and **b** ^{13}C NMR spectra of 1-Heptyl 2-octylcyclohexane-1,2-dicarboxylate.

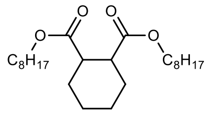

^{1}H NMR (400 MHz, DMSO-d^6): δ 4.03-3.91 (m, 4H), δ 2.82-2.81 (m, 2H), 2.02-1.79 (m, 2H), 1.65-1.27 (m, 24H), 0.89-0.85 (12H, m) ppm, (cis:trans = 0: ~100). ^{13}C NMR (100 MHz, DMSO-d^6): δ 173.93, 66.85, 66.83, 42.83, 38.88, 30.57, 30.55, 29.07, 29.06, 26.55, 24.01, 23.96, 23.93, 23.12, 14.18, 11.12 ppm.

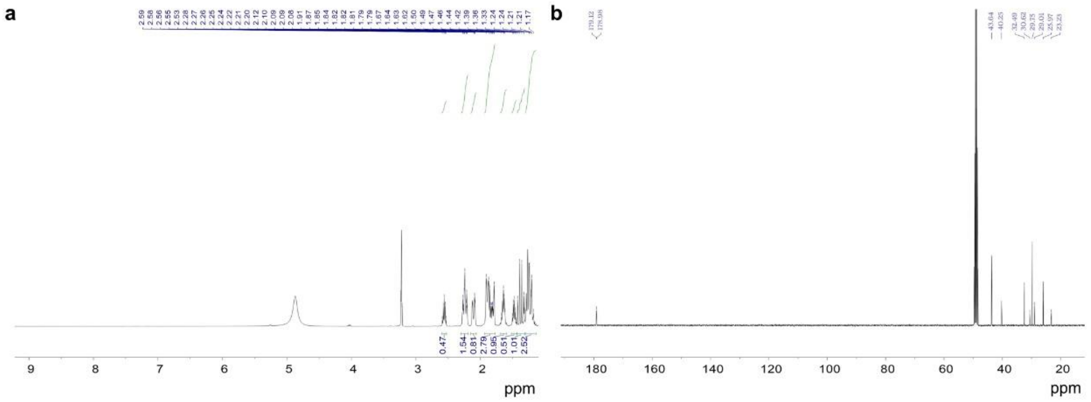

Supplementary Figure 24. **a** ^{1}H NMR and **b** ^{13}C NMR spectra of Cyclohexane-1,3-dicarboxylic acid.

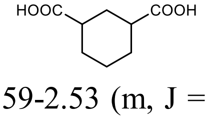

^{1}H NMR (400 MHz, MeOD): δ 2.59-2.53 (m, J = 6 Hz, 0.47H), 2.24 (tt, J = 12 ,3.2 Hz, 1.54H), 1.91-1.79 (m, 3H), 1.67-1.62 (m, 1H), 1.50-1.17 (m, 4H) ppm, (cis:trans = 77:23). ^{13}C NMR (100 MHz, MeOD):δ 179.12, 178.98, 43.64, 40.25, 32.49, 30.62, 29.75, 29.01, 25.97, 23.23 ppm.

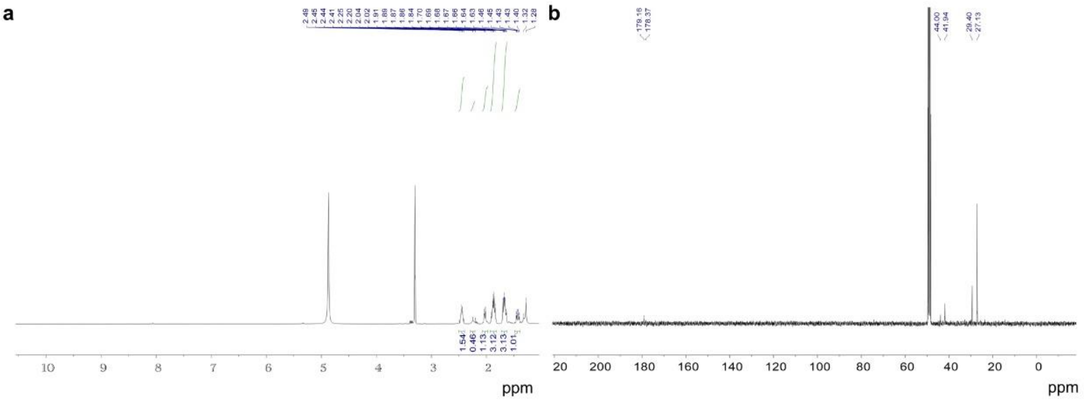

Supplementary Figure 25. **a** ^{1}H NMR and **b** ^{13}C NMR spectra of Cyclohexane-1,4-dicarboxylic acid.

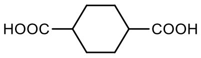

^{1}H NMR (400 MHz, MeOD): δ 2.49-2.20 (m, 2H), 2.04-2.02 (m, 1H), 1.91-1.84 (m, 3H), 1.70-1.63 (m, 3H), 1.46-1.40 (m, 1H) ppm, (cis:trans = 77:23). ^{13}C NMR (100 MHz, MeOD): δ 179.16, 178.37, 44.00, 41.94, 29.40, 27.13 ppm.

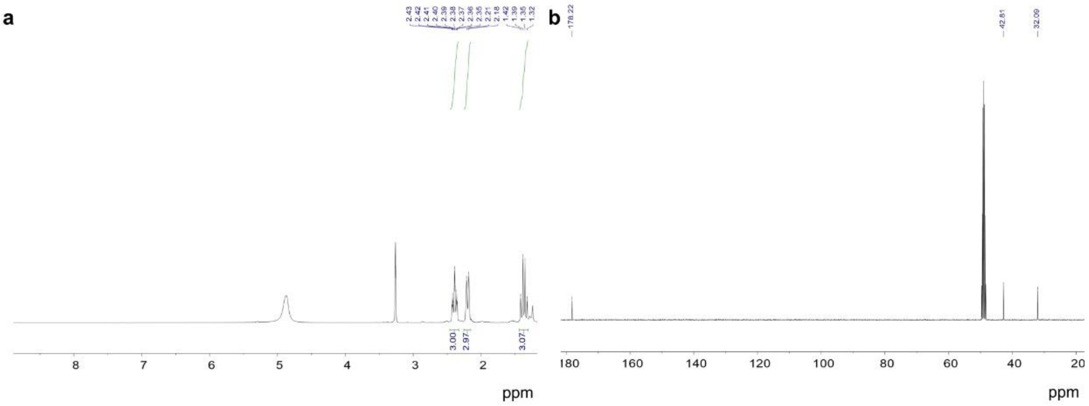

Supplementary Figure 26. **a** ^{1}H NMR and **b** ^{13}C NMR spectra of Cis-cyclohexane-1,3,5-tricarboxylic acid.

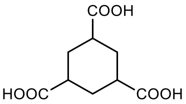

^{1}H NMR (400 MHz, MeOD): δ 2.39 (tt, J = 12.4, 3.2 Hz, 3H), 2.21-2.18 (d, J = 12.4 Hz, 3H), 1.42-1.32 (q, J = 12.8 Hz, 3H) ppm, (cis:trans = ~100:0). ^{13}C NMR (100 MHz, MeOD): δ 178.22, 42.81, 32.09 ppm.

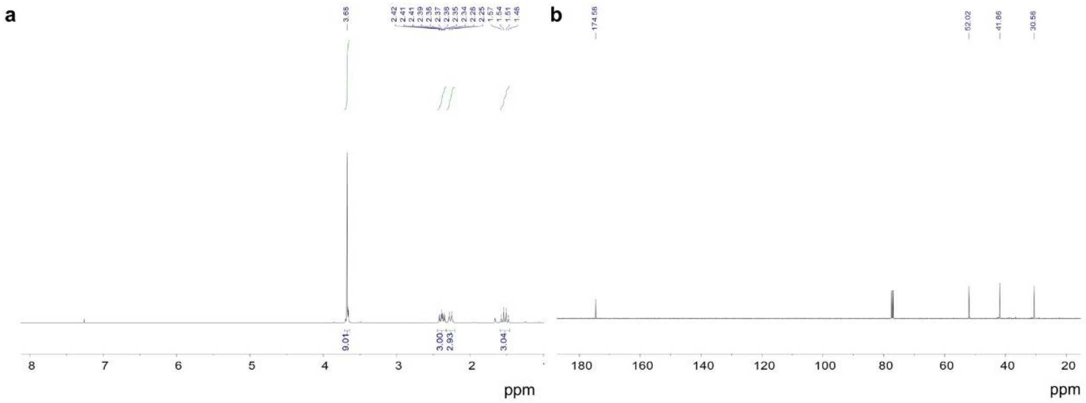

Supplementary Figure 27. **a** ^{1}H NMR and **b** ^{13}C NMR spectra of Cis-cyclohexane-1,3,5-tricarboxylic acid trimethyl ester

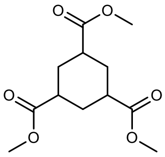

^{1}H NMR (400 MHz, $CDCl_3$): δ 3.68 (s, 9H), 2.38 (tt, J = 12.4, 3.2 Hz, 3H), 2.28-2.25 (d, J = 12.4 Hz, 3H), 1.57-1.48 (q, J = 12.8 Hz, 3H) ppm, (cis:trans = ~100:0). ^{13}C NMR (100 MHz, CDCl3): δ 174.58, 52.02, 41.86, 30.56 ppm.

Supplementary Table 1. The catalytic results of Ru/TiO_2 and Pd/TiO_2 in the BA hydrogenation.

Cat.	Conv. (%)	Sel. (%)
Ru/TiO_2	~0	--
Pd/TiO_2	~0	--

Reaction conditions: 40 °C, 10 bar H_2, 1 h, S/C = 700, 3 mL n-hexane, 2h. Selectivity to CCA.

Supplementary Table 2. The summarized results of BA hydrogenation under mild conditions in literatures.

Catalyst	Conditions	TOF (h^{-1})[a]	Ref.
Pd/CN	85 °C, 1 bar H_2, H_2O	0.883	9
Pd/AC	85 °C, 15 bar H_2, H_2O	5.7	10
Pd/CNF	85 °C, 15 bar H_2, H_2O	0.35	11
Pd/MCN	85 °C, 25 bar H_2, H_2O	26	12
Pd/CNT	100 °C, 35 bar H_2, H_2O	87	13
Pd/UiO-66-2OH	85 °C, 1 bar H_2, H_2O	0.416	14
Pd/800N-AC	110 °C, 25 bar H_2, H_2O	35	15
Ru/C-TiO_2	70 °C, 25 bar H_2, H_2O	45	16
Ru/NFPC	55 °C, 10 bar H_2, H_2O	69.8	17
Ru/C	60 °C, 5 bar, iPrOH	0.36	18
Rh/N-C-700	55 °C, 10 bar H_2, H_2O	320	19
Rh/C	50 °C, 20 bar H_2, 100 bar CO_2	45	20
Pt NWs	60 °C, 1 bar H_2, H_2O	2.03	21
Pt NWs	70 °C, 10 bar H_2, acetic acid	5.3	22
Ir/CN	55 °C, 10 bar H_2, H_2O	57	23
RuPd/CN	85 °C, 1 bar H_2, H_2O	2.2	24
Pt/TiO_2	40 °C, 10 bar H_2, hexane	638 (2200)[b]	This work
Pt/TiO_2	80 °C, 50 bar H_2, hexane and acetic acid	4490 (15480)[b]	This work
Pd/C	80 °C, 50 bar H_2, hexane and acetic acid	~11	This work
Ru/C	80 °C, 50 bar H_2, hexane and acetic acid	94	This work

[a]Apparent TOF; [b]Data in parentheses was normalized by CO chemisorption.

Supplementary Table 3. Pt/TiO_2 catalyzed BA hydrogenation with different solvents.[a]

Solvent	Conv. (%)	Sel. (%)[b]	p[c]	δ[d]
n-hexane	> 99	> 99	0.009	4.98
H_2O	84	> 99	1.000	0.792
cyclohexane	78	> 99	0.006	3.84
Acetic acid	68	96	0.648	2.61
isopropyl alcohol	34	98	0.546	3.48
Ethyl acetate	30	97	0.228	3.53
EtOH	19	96	0.654	3.66
Tetrahydrofuran	10	89	0.207	3.37
Acetone	4	93	0.355	3.12

[a]Reaction conditions: BA = 0.12 mmol, S/C = 250, 3 mL solvent, 40 °C and 10 bar H_2, 1 h. [b]Selectivity to CCA. [c]Relative polarity. The values are normalized from measurements of solvent shifts of absorption spectra and were extracted from Christian Reichardt, Solvents and Solvent Effects in Organic Chemistry, Wiley-VCH Publishers, 3rd ed., 2003. [d]Solubility of H_2 (10^{-3} mol L^{-1}, taken from ref. 2, 298.15 K, 1 atm H_2).

Supplementary Table 4. Reaction orders for BA hydrogenation with Pt/TiO_2 and Pt/TiO_2-450.

Reaction rate: $r = k[BA]^{\alpha}[H_2]^{\beta}$

Cat.	BA order (α)	H_2 order (β)	Overall (α+β)
Pt/TiO_2	-0.34	+0.54	+0.20
Pt/SiO_2-450	+0.45	+0.02	+0.47

Supplementary Table 5. The H_2-D_2 exchange results of Pt/TiO_2 catalysts.

Cat.	Conv. (%)	Normalized activity
Pt/TiO_2	5.96	100
Pt/TiO_2-200	1.37	23
Pt/SiO_2-450	0.67	11

Reaction conditions see experimental section

Supplementary References

1. Chambers, R. P. & Boudart, M. Lack of Dependence of conversion on flow rate in catalytic studies, *J. Catal,* **6**, 141-145 (1966).

2. Young, C. L. *Hydrogen and deuterium* (ed. Young, C. L.) volume 5/6 (Oxford, 1981).

3. Chaudhari, R. V., Gholap, R. V., Emig, G. & Hofmann, H. Gas-liquid mass transfer in "dead-end" autoclave reactors. *Can. J. Chem. Eng.* **65**, 744- 751 (1987).

4. Sano, Y., Yamaguchi, N. & Adachi, T. Mass transfer coefficients for suspended particles in agitated vessels and bubble columns. *J. Chem. Eng. Jpn.* **7**, 255-261 (1974).

5. Stephens, K. J. et al. A mechanistic study of polyol hydrodeoxygenation over a bifunctional Pt-WO_x/TiO_2 catalyst, *ACS Catal.* **10**, 12996−13007 (2020).

6. Sporka, K., Hanika, J., Růžička, V. & Halousek, M. Diffusion of gases in liquids. III. Diffusion coefficients of hydrogen in organic solvents. *Collect. Czech. Chem. Commun.* **36**, 2130-2136 (1971).

7. Singh, R. P. & Sinha, C. P. Viscosities and activation energies of viscous flow of the binary mixtures of n-hexane with toluene, chlorobenzene, and 1-hexanol. *J. Chem. Eng. Data* **29**, 132-135 (1984).

8. Bates, R. L., Fondy, P. L. & Corpstein, R. R. An examination of some geometric parameters of impeller power, *Ind. Eng. Chem. Process Des. Dev.* **2**, 310–314 (1963).

9. Xu, X. et al. Hydrogenation of benzoic acid and derivatives over Pd nanoparticles supported on N-doped carbon derived from glucosamine hydrochloride. *ACS Catal.* **4**,

3132-3135 (2014).

10. Anderson, J. A. et al. Aqueous phase hydrogenation of substituted phenyls over carbon nanofibre and activated carbon supported Pd. *J. Catal.* **270**, 9–15 (2010).

11. Anderson, J., McKenna, F. M., Linares-Solano, A. & Wells, R. K. Use of water as a solvent in directing hydrogenation reactions of aromatic acids over Pd/carbon nanofibre catalysts. *Catal. Lett.* **119**, 16-20 (2007).

12. Jiang, H. et al. Selective hydrogenation of aromatic carboxylic acids over basic N-doped mesoporous carbon supported palladium catalysts. *Appl. Catal. A Gen.* **520**, 73-81 (2016).

13. Hu, Y., Chen, W., Xie, X. & Song, W. Carbon nanotubes-supported well-dispersed Pd nanoparticles for the efficiently selective hydrogenation of benzoic acid to synthesize cyclohexane carboxylic acid. *Nano* **14**, 1950008 (2019).

14. Chen, D., Yang, W., Jiao, L., Li, L. & Jiang, H. L. Boosting catalysis of Pd nanoparticles in MOFs by pore wall engineering: the roles of electron transfer and adsorption energy. *Adv. Mater.* 2000041 (2020).

15. Nie, R., Jiang, H., Lu, X., Zhou, D. & Xia, Q. Highly active electron-deficient Pd clusters on N-doped active carbon for aromatic ring hydrogenation. *Catal. Sci. Technol.* **6**, 1913-1920 (2016).

16. Zhang, H., Li, G., Nie, R., Lu, X. & Xia, Q. One-pot synthesized mesoporous C-TiO_2 hybrid for Ru catalyzed low-temperature hydrogenation of benzoic acid. *J. Mater. Sci.* **54**, 7529–7540 (2019).

17. Li, M., Tang, M., Deng, J. & Wang, Y. Nitrogen-doped flower-like porous carbon

materials directed by in situ hydrolysed MgO: promising support for Ru nanoparticles in catalytic hydrogenations. *Nano Res.* **9**, 3129-3140 (2016).

18. Maegawa, T. et al. Efficient and practical arene hydrogenation by heterogeneous catalysts under mild conditions. *Chem.-Eur. J.* **15**, 6953-6963 (2009).

19. Cao, Y. et al. In situ synthesis of chitin-derived Rh/N–C cataylsts: efficient hydrogenation of benzoic acid and derivatives. *ACS Sustainable Chem. Eng.* **5**, 9894–9902 (2017).

20. Wang, H. & Zhao, F. Catalytic ring hydrogenation of benzoic acid with supported transition metal catalysts in $scCO_2$. *Int. J. Mol. Sci.* **8**, 628–634 (2007).

21. Yu, T., Wang, J., Li, X., Cao, X. & Gu, H. An improved method for the complete hydrogenation of aromatic compounds under 1 bar H_2 with platinum nanowires, *ChemCatChem* **5**, 2852-2855 (2013).

22. Guo, Z., Hu, L., Yu, H., Cao, X. & Gu, H. Controlled Hydrogenation of Aromatic Compounds by Platinum Nanowire Catalysts. *RSC Adv.* **2**, 3477-3480 (2012).

23. Tang, M. et al. Highly effective Ir-based catalysts for the benzoic acid hydrogenation: experiment and theory guided catalysts rational design. *Green Chem.* **19**, 1766-1774 (2017).

24. Tang, M. et al. RuPd alloy nanoparticles supported on N-doped carbon as an efficient and stable catalyst for benzoic acid hydrogenation. *ACS Catal.* **5**, 3100–3107 (2015).
