# Supplementary material for: Hydrogenation of benzoic acid derivatives over Pt/TiO2 under mild conditions
Source: Commun Chem. 2021 Apr 16;4:54. doi: 10.1038/s42004-021-00489-z (PMC9814562; doi:10.1038/s42004-021-00489-z)
Supplement: Supplementary file 2 — Description of Additional Supplementary Files [file 42004_2021_489_MOESM2_ESM.pdf]

Description of Additional Supplementary Files

File Name: Supplementary Data 1
Description: the optimized fractional coordinates for different adsorbates on Pt(111)/Pd(111)/Ru(1000) surface.
